# A New Endophytic Fusarium Oxysporum Gibberellic Acid: Optimization of Production Using Combined Strategies of Experimental Designs and Potency on Tomato Growth under Stress Condition

**DOI:** 10.1155/2020/4587148

**Published:** 2020-03-12

**Authors:** Marwa Ben Rhouma, Mouna Kriaa, Yosri Ben Nasr, Lotfi Mellouli, Radhouane Kammoun

**Affiliations:** ^1^Laboratoire de Microorganisme et de Biomolécules, Centre de Biotechnologie de Sfax, Université de Sfax, Route de Sidi Mansour Km 6, B.P. 1177, 3018 Sfax, Tunisia; ^2^Institut Supérieur de Biotechnologie de Sfax, Route de Sokra Km 3, B.P. 261, 3000 Sfax, Tunisia

## Abstract

This study reports the potential of the endophytic fungi identified as a *Fusarium oxysporum* to produce gibberellic acid (GA3). The GA3 production was confirmed by high performance liquid chromatography. To improve the production of this phytohormone under solid state fermentation (SSF), successive optimization strategies were used. Firstly, Plackett-Burman design was applied for screening medium components and culture condition. Under the optimized condition, GA3 yield (7.14 g/kg) was 2.62-fold higher than by the use of the initial condition (2.72 g/kg). The concentration of the most influential parameters and their interaction were optimized with a Box-Behnken experimental design. The optimized condition led to a 1.14-fold enhancement in GA3 production, reaching 8.16 g/kg. The GA3 crude extract obtained by SSF was then used to study its ameliorative role on adverse salinity effect on tomato plants (*Solanum lycopersicum* L.). The interactive effects of different GA3 concentrations were examined on morphological and physiological parameters of tomato plants. The application of GA3 (10^−6^ M) under salt stress condition (100 mM) was found to improve growth and physiological parameters including plant height, total chlorophyll, starch, and proline contents. The exogenous application of GA3 is a potent strategy to reverse abiotic stress that affect the agricultural productivity and limit plant growth and yield.

## 1. Introduction

Salinity is one of the major agricultural productivity limiting factors around the world. It seems to disturb the majority of plant's metabolic processes, such as growth, photosynthesis, and biosynthesis of compatible solutes. Thus, low precipitation, surface evaporation, irrigation with poor water quality, and some agricultural practices are among the main causes of salinity. Each year, around 20 million ha of field is lost due to this phenomenon [1]. Recently, more than 800 million ha are affected. In Tunisia, this problem is faced in around 1.5 million ha [2].

To cope with the effects of salinity, plants develop physiological and biochemical mechanisms assuring favorable water balance to maintain crop development. But in the most cases, intervention is crucial to counteract the stress effects.

Alternative strategies are being tried. In the past, scientists generally focused on the association between endophytic microorganisms and their host plants. These particular fungi have been reported for their ability to enhance plant growth and alleviate biotic and abiotic stress [3, 4]. Actually, researchers are rather interested in their yield (phytohormones and enzymes) and especially their applications. In fact, exogenous application of phytohormones such as gibberellins is well known to improve abiotic stress tolerance in crops [5, 6]. For instance, gibberellins foliar spray counteracted the growth restriction caused by NaCl in wheat, rice, and tomato plants. The exogenous application of gibberellins plays a critical role in adjusting osmotic potential and sustaining plant growth under salt stress. Gibberellic acid (GA3) belongs to the family of gibberellins and it is known as an important plant growth regulator, stimulating numerous development processes. GA3 is a class of diterpenoids with a high industrial value which involves agriculture, nurseries, tea gardens, etc [7–9]. The use of this phytohormone as a plant growth regulator was extremely limited due to its scarcity and high costs (25$/g) in the market.

Generally, gibberellin industrial production is performed in submerged fermentation. However, the production cost is extremely high due primarily to elevated energy consumption and very low yield. Recently, the use of solid state fermentation (SSF) has gained a lot of attention [10]. This process simulates the biology of filamentous fungi, offering many advantages when compared to the submerged fermentation (SMF), such as higher productivity, simplicity, and lower downstream costs. Thus, SSF was considered as the best alternative to SMF techniques in the production of secondary metabolites and in the valorization of agro-resources [11, 12].

In this context, we studied the effect of a new isolated *Fusarium oxysporum* GA3 on the physiological, morphological, and biochemical responses of tomato plants under salt stress conditions. In this work, we have focused to optimize GA3 production under SSF on low-cost agro-industrial waste as substrates using statistical experimental designs.

## 2. Material and Methods

### 2.1. Isolation and Screening of Endophytic Fungi for GA3 Production

In order to screen GA3-producing fungi, 23 new isolates were isolated from the root of the *Coriandrum sativum* plant. Roots were cut into small pieces and washed with autoclaved distilled water. The samples were dipped into 4% sodium hypochlorite for 60 seconds and 70% ethanol for 5 seconds. The surface sterilized roots were washed with sterile water and left to dry under sterile conditions. The samples were inoculated on potato dextrose agar (PDA) (39 g. L^−1^) plates amended with 50 mg/L tetracycline to abolish the bacterial growth and incubated at 28–30°C for 3 to 4 days. The new fungi were isolated from the plates and subcultured on PDA. Subculture was continued until pure isolates were obtained. The isolates were screened for the GA3 production. The strains were harvested from the plates, dislodged under aseptic conditions, and then tested on a Czapek medium (composed of (g/L) sucrose, 30; NaNO_3_, 3; K2HPO_4_, 1; MgSO_4_.7H_2_O, 0.5; KCl, 0.5; and FeSO_4_, 0.01 at pH 6) for 8 days at 30°C and 200 rpm. Fungi strains with potential GA3 production were retained for further study.

### 2.2. Molecular Identification of the Selected Fungi

Molecular biology techniques were carried out as described elsewhere [13]. The B28 strain was cultivated on a Czapek medium at 30°C under continuous agitation at 200 rpm. Mycelia from 48-h cultures were harvested and DNA was extracted.

The internal transcribed spacer regions (ITS) were submitted to PCR amplification using fungus-specific primers, namely ITSl (5′-TCC GTA GGT GAA CCT GCG G-3′) and ITS4 (5′-TCC TCC GCT TAT TGA TAT G-3′). The amplification was initiated by incubating the PCR reaction mixture at 95°C for 5 min, followed by 35 cycles of denaturation for 30 s at 94°C. The reaction was annealed at 50°C for 30 s and terminated with extension consisting of 1 min at 72°C and final steps of 10 min at 72°C. The PCR products were analyzed on an agarose gel (1%) and purified by the Polyethylene Glycerol- (PEG-) NaCl method. The nucleotide sequences were determined using the Big-Dye Terminator v3.1 Cycle Sequencing Kit and the automated ABI Prism1 3100-Avant Genetic Analyser (Applied Biosystems). The sequence obtained for the intergenic region rRNA. The BLAST search program (http://www.ncbi.nlm.nih.gov/BLAST/) was used to look for nucleotide sequence homology.

### 2.3. Submerged Fermentation

*F. oxysporum* strain B28 culture was inoculated from the PDA slants into 250 ml of Czapek medium (composed of (g/L) sucrose, 30; NaNO_3_, 3; K2HPO_4_, 1; MgSO_4_.7H_2_O, 0.5; KCl, 0.5; and FeSO_4_, 0.01 at pH 6) and incubated at 30°C for 10 days at 150 rpm. Separation of fungal mycelium and broth by filtration through Whatman No.1 filter paper. The filtrate was then centrifuged and the supernatant was used for GA estimation.

### 2.4. Estimation of Gibberellic Acid Production

GA3 was extracted and estimated by the method of Holbrook and Bailey [14]. The filtered fermented Czapek medium (10 ml) was transferred to a centrifuge tube and added by 0.5 ml zinc acetate (1 M) solution and shaken for 3 min. This mixture was then supplemented by 0.5 ml of potassium ferrocyanide solution (1 M) and centrifuged for 15 min. 2.5 ml of supernatant was transferred to a 250 ml flask containing 8 ml absolute ethanol and 90 ml HCl (30%). For control, 35 ml HCl solution (5%) was taken in a 250 ml flask and the volume made to 100 ml with 65 ml of distilled water. The flasks were incubated at 20 ± 2°C in a water bath for 75 min and the absorbance was read at 254 nm. The GA3 concentration was obtained from a standard GA curve. This standard curve was established from known solutions of pure gibberellic acid (obtained from Sigma Aldrich, Germany) and a linear relationship was established throughout a concentration range of 0 to 0.4 g.L^−1^.

### 2.5. Extraction and Identification of Gibberellic Acid

The filtrate of the fermentation broth was acidified to pH 2-2.5 with 18% HCl and extracted twice with ethyl acetate. Sixty percent methanol (MeOH) with a minimum volume was added and the pH was adjusted up to 8 ± 0.3 by adding 2 N NaOH. After filtration, GA3 was added to the filtrate as an internal standard. The quantification of GAs was performed by high performance liquid chromatography (HPLC-UV, Agilent 1260 Series System; Bio-Rad C18 column; G1315D1260 DAD VL series number DEAAX03593; with a mobile phase of methanol and water (80 : 20), flow rate of 20 *μ*l/min, and column temperature of 25°C. The detection took place at 210 nm [15].

### 2.6. Solid State Fermentation

As substrates for fermentation, wheat bran, barley bran, oat bran, and sesame bark were obtained from local producers. Then, carbon sources were air-dried and milled to 0.1–0.25 mm particle size. For preliminary experiment, cultures were carried out in initial condition 500-ml Erlenmeyer flasks containing 10 g agricultural residues, 3 g sucrose, 0.3 g NaNO_3_, 0.1 g K_2_HPO_4_, 0.05 g MgSO_4_.7H_2_O, 0.05 g KCl, and 0.001 g FeSO_4_. To reach the desired moisture level (70%), an appropriate volume of distilled water was added into flasks. Flasks were autoclaved, cooled, and inoculated with 10 ml of fungi inoculums, which were grown on Czapek medium at 200 rpm at 30°C for 4 days. Flask content was well mixed and incubated at 30°C for 8–10 days. The production of GA3 was evaluated separately in these substrates.

### 2.7. Optimization of Enzyme Production under SSF

The Plackett–Burman design, an efficient way for the screening of a large number of variables [16], was employed for choosing medium component and fermentation parameters that enhanced the GA3 production. All independent variables were tested at two levels (-) and (+), which referred to high and low, respectively. In this study, the additional nutrients were screened by a Plackett–Burman design for thirteen variables at two levels. The thirteen assigned variables were screened in 16 experimental designs. GA3 yields were taken as response (Table 1). The Taguchi methodology was used to understand the relationship between factors of medium components, adjust their concentrations, and reduce their number for GA3 production by the entophytic fungi *F. oxysporum*. In this study, six factors at three levels of variations (Table 2) were used in these experiments. The selected variables, including sesame bark, wheat straw, date waste, NaNO_3_, urea, and (NH_4_)_2_SO_4_ because they have significant impact on GA3 production as screened by Plackett–Burman design, were tested by Taguchi design (Table 3). The combination of factors and levels, for all the experiments shown in (Table 4), were in conformity with Taguchi's L25 orthogonal array. A validation test was also conducted to check the optimum condition.

Thereafter, the Box-Behnken Response Surface Methodology Design was performed to determine the level of each of these factors giving the highest GA3 production. Modelling was achieved using a second-order polynomial equation:

(1) $$\begin{array}{c} y=b0+\overset{n}{\underset{i=1}{\sum }}b_{i}x_{i}+\overset{n}{\underset{i=1}{\sum }}b_{ii}x_{i}^{2}+\overset{n-1}{\underset{i=j}{\sum }}\overset{n}{\underset{j=i+1}{\sum }}b_{ij}x_{ij}, \end{array}$$

where *y* is the GA3 activity, *b*0 is the offset term, *b*_*i*_ is the linear effect, *b*_*ii*_ is the squared effect, *b*_*ij*_ is the first-order interaction effect, and *X*_*i*_ is the independent variable.

Regression analysis of the experimental data yielded the following quadratic model equation:

(2) $$\begin{array}{c} Y=0.928+0.384*X1+7.811*X2+25.502*X3+1.611*X1X2-0.856*X1X4-69.995*X3X3-2.749*X4X4, \end{array}$$

where *Y* is the GA3 production (g/kg ds) and *X*1, *X*2, *X*3, and *X*4 are, respectively, date waste, NaNO_3_, urea, and (NH_4_)_2_SO_4_.

### 2.8. Experimental Design

Trials were conducted at the Biotechnology Center of Sfax, Tunisia. Tomato plants with three leaves (*Solanum lycopersicum L.*) were transplanted into 5 l pots filled with soil and plant ash (50 : 50). The pots were kept under ambient environment conditions with natural sunlight and temperature (from March to July, 2017). Air temperature ranged between 21 ± 2.5 and 27.5 ± 3.5°C. Relative humidity varied from 56 ± 5.5 to 70 ± 6.5.

All plants were divided into 9 groups according to the following treatments:

*CP*: control plants untreated with NaCl and irrigated with tap water

*SSP1*: stressed plants treated with tap water containing 50 mM of NaCl

*SSP2*: stressed plants treated with tap water containing 100 mM of NaCl

*SSP1+GA31*: stressed plants treated with tap water containing 50 mM of NaCl+ foliar spray application of gibberellic acid GA3 (10^−5^ M)

*SSP1+GA32*: stressed plants treated with tap water containing 50 mM of NaCl+ foliar spray application of GA3 (10^−6^ M)

*SSP1+GA33*: stressed plants treated with tap water containing 50 mM of NaCl+ foliar spray application of GA3 (10^−7^ M)

*SSP2+GA31*: stressed plants treated with tap water containing 100 mM of NaCl+ foliar spray application of GA3 (10^−5^ M)

*SSP2+GA32*: stressed plants treated with tap water containing 100 mM of NaCl+ foliar spray application of GA3 (10^−6^ M)

*SSP2+GA33*: stressed plants treated with tap water containing 100 mM of NaCl+ foliar spray application of GA3 (10^−7^ M)

### 2.9. Plant Growth Measurement

At the end of each experiment, growth parameters (plant height, number of leaves per plant, and number of fruit per plant) were determined. Plant height was measured using a meter rule (cm) from the base of the plant to the apical region of the leaf. At harvest, plants were divided into roots and leaves and washed in distilled water. For the determination of fresh weight (FW), samples were dried on filter paper and weighed. Samples of leaves were either immediately used for analyses. Other samples of leaves were oven-dried at 70°C to a constant weight as described previously by Zouari et al. [17]. Finally, the oven-dried plant materials were ground in a grinder.

### 2.10. Chlorophyll Content

For chlorophyll a, b, and total chlorophyll analyses, fresh leaves were incubated in the dimethyl formamide in obscurity at 4°C for one week. In the end of this period, foliar pigment contents were determined spectrophotometrically according to the method of Arnon [18]. Absorbance was read at 645 nm (*A*_645_), 652 nm (*A*_652_), and 663 nm (*A*_663_) using Helios *β* spectrophotometer (ThermoSpectronic, Courtaboeuf, France). Concentrations of chlorophyll a, chlorophyll b, and total chlorophyll were determined by the following equations:

(3) $$\begin{array}{c} \text{Chlorophyll a }\left(\mu \text{g}/\text{ml}\right)=0.0127\times A_{663}‐0.00269\times A_{645},\\ \text{Chlorophyll b }\left(\mu \text{g}/\text{ml}\right)=0.0229\times A_{645}‐0.00468\times A_{663},\\ \text{Total chlorophyll}\left(\mu \text{g}/\text{ml}\right)=A_{652}/34.5. \end{array}$$

### 2.11. Soluble Sugars and Starch Contents

Soluble sugar contents were determined according to the method of McCready et al. [19]. Dry powder samples (100 mg) were mixed with 10 ml of ethanol 80% in covered glass tubes and boiled at 70°C for 20 min. After cooling, 250 *μ*l of the extract was mixed with 5 ml cold anthrone reagent (200 mg of anthrone dissolved in 100 ml of cold 95% H_2_SO_4_). After agitation, the reagent mixture was boiled at 95°C for 10 min. After cooling, the absorbance was read at 640 nm. Soluble sugar concentration was calculated using glucose solutions to develop a standard curve.

Starch contents were also determined according to the method of McCready et al. [19]. Dry powder samples (100 mg) were extracted in boiling 80% ethanol. The residue left behind after alcoholic extraction was dissolved in 5 ml of 52% perchloric acid for 1 h. The mixture was filtered and made up to 100 ml with distilled water. An aliquot of 1 ml of the extract was mixed with 4 ml of distilled water and 10 ml of cold anthrone reagent (200 mg of anthrone dissolved in 100 ml of cold 95% H_2_SO_4_) and boiled at 100°C for 10 min. After cooling, the absorbance was read at 630 nm. Starch concentration was calculated using glucose solutions to develop a standard curve.

### 2.12. Proline Content

Proline content was determined according to Bates et al. [20]. One gram of fresh leaves was homogenized with a mortar and pestle with 10 ml of 3% aqueous sulfosalicylic acid. The homogenate was filtered through Whatman No 1 filter paper and the residue was reextracted. The extracts were pooled and made up to 10 ml with aqueous sulfosalicylic acid and used for estimation. An aliquot of 2 ml of the extract was mixed with 2 ml of glacial acetic acid and 2 ml ninhydric acid and boiled at 100°C for 1 h. After cooling, 2 ml of toluene were added to the mixture. The chromophore-containing toluene was separated, and the absorbance was measured at 520 nm with a UV/vis spectrophotometer. Toluene was used as a blank, and proline content was calculated using L-proline for the standard curve.

### 2.13. Ion Concentrations

The leaves used for ion determinations were washed with distilled water, oven dried at 70°C for 72 h, and ground to a fine powder. One gram of the powder was placed at 250°C for 3 h in an oven and then transferred to 100 ml of dilute nitric acid (1 M). Na^+^, K^+^, and Ca^2+^ concentrations were determined using a flame photometer (Jenway, PEP-7). For chloride determination, dry plant material was extracted with 40 ml HNO3 (0.2 N).

### 2.14. Statistical Analysis

One-way analysis of variance (ANOVA) was used. All analyses were done using the SPSS program (V23.0) by Tukey's post hoc test to determine the significant differences between treatments. The results were expressed as mean values and standard errors from the three replications. All tests were performed at a 0.05 level of significance.

## 3. Results and Discussions

### 3.1. Isolation, Screening, and Identification of GA3-Producing Fungi Strain

**Figure 1:**
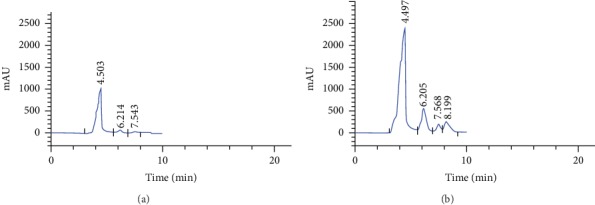
HPLC-RID chromatogram of the fermented extract and GA3 standard (reagent grade). (a) Analysis of GA3 standard solution at 0.100 g.L^−1^ and (b) fermented extract contents GA3 according the conditions used in the present study.

Maximum GA3 production was achieved by the strain B28 (0.371 g/l). Consequently, this fungal strain was selected for further studies as the most potent isolate for GA3 production. The culture filtrate of fungal strain was subjected to chromatography analysis for the determination of GA3. The results showed that the culture filtrate of strain B28 contained GA3 (Figures 1(a) and 1(b)).

The ITS amplification of the strain by PCR resulted in a product of 528 bp in size. The PCR-amplified ITS sequence from B28 was determined (GenBank accession no. MN816007) and was 99% similar to that of *Fusarium oxysporum*. Therefore, the strain was identified as B28 strain *Fusarium oxysporum*.

### 3.2. Optimization of GA3 Production

#### 3.2.1. Screening of the Main GA3 Production Factors

The obtained result (Table 1) showed a wide variation of GA3 production ranging from 0.89 g/kg ds to 4.27 g/kg ds. This variation illustrates the importance of this step to screen the most influential variable and the level of the others [13]. The analysis of the contrast coefficient (b) indicated that sesame bark, wheat straw, date waste, NaNO_3_, urea, and (NH_4_)_2_SO_4_ displayed a positive effect on GA3 production; whereas inoculums size, sucrose, molasses, and fish meal had a negative effect on it. The critical effect of these variables on GA3 production was confirmed by statistical analyses, particularly by *t* test and *P* value (Table 2). The variables having the most important contrast coefficients including NaNO_3_, urea, sesame bark, (NH_4_)_2_SO_4_, and date waste were the most significant variables affecting GA3 production by statistical analysis. These factors presented a very low *P* value and the highest level of significance with a *t* value of 2.75, 2.21, 2.012, 1.902, and 1.87, respectively, demonstrating their pronounced effect on GA3 production. The present study is the first contribution towards the use of that sesame bark, wheat straw, and date waste, which are agro-wastes and readily available complex substrates as a complex organic source for the production of GA3 by B28 strain *Fusarium oxysporum.*

### 3.3. Optimization of GA3 Production by the Taguchi Method

The Taguchi method was successfully used to examine the effects of different medium components such as carbon and nitrogen sources influencing the production of various microbiology metabolites [21–24].

In this study, the L25 Taguchi design was used to determine the relative effect of the selected factors (sesame bark, wheat straw, date waste, NaNO_3_, urea, and (NH_4_)_2_SO_4_ on GA3 production by B28 strain *Fusarium oxysporum* and also to choose the most adequate substrate among the studied ones. The analysis of the results showed that the GA3 production strictly depends on the nutritional medium components corresponding to the combined effect of the examined parameters over their defined ranges. Indeed, a huge variation in production yield ranging from 1.72 g/g of substrate (run 17) to 6.35 g/g of substrate (run 21) was observed (Table 3). This variation revealed the relevance of this approach to choose the best influential variable and the level of each factor. Table 4 shows that GA3 production is mainly influenced by the concentration of (NH_4_)_2_SO_4_ which represents the largest contribution compared with the other factors (29%). This critical factor causes the greatest response in GA3 production at level 2. Sesame bark and NaNO_3_ also presented an important contribution of about 17 and 18%, respectively. Maximum response occurred at level 2 and level 4, respectively. The results also showed that GA3 production in SSF is evenly influenced by the other tested variables (wheat straw, urea, and date waste) which represent significant contributions greater than 10%. Wheat straw has a larger effect on the GA3 production yield in level 4 whereas urea and date waste have the greatest effect at level 5. This result proves the importance of our optimization strategy. Indeed, the Taguchi design was successfully applied to determine the relative importance of the preselected factors and to select the suitable solid substrate for GA3 production in solid state fermentation. It is well documented that solid state fermentation processes are largely influenced by the nature of the solid substrate. The substrates are generally water insoluble polymers of cellulosic or starchy material [25]. Our study indicated that GA3 production was increased by the addition of different compounds as inducers to the media. Therefore, GA3 production increased by the use of date waste and wheat straw at its higher levels. Several studies have reported the production of GA3 in SSF using waste and byproducts [9, 10, 24]. Panchal and Desai [26], who investigated the production of GA3 of *Fusarium moniliforme* in SSF, used the commercial wheat bran (CWB) mineral salt acid as a solid substrate. They reported that the increase of GA3 production may be in direct correlation with the complexity of the carbon sources. Citric pulp was largely used as substrate/support for GA3 production in SSF [24, 27–29]. Nevertheless, there were no earlier reports using date waste and wheat straw as carbon source and solid substrate for GA3 production in SSF. Thus, this study may be the first report to note that date waste and wheat straw were potential substrates for GA3 production. It was interesting to note that soluble cellulose and hemicellulose fractions of wheat byproduct served as potent carbon sources which led to a sufficient carbon and nitrogen ratio for efficient metabolites production [21, 23, 30]. Date waste was investigated as an excellent growth substrate for the production of glucose oxidase in SSF [23].

The validity of the optimum conditions (7.5 g/flask sesame bark, 12.5 g/flask wheat straw, 12.5 g/flask dates waste, 1 g/flask NaNO_3_, 0.5 g/flask urea, and 0.5 g/flask NH_4_NO_3_) showed an enhanced GA3 yield of 7.14 g/kg ds, which was 2.63-fold higher than at the initial conditions (2.72 g/kg ds).

### 3.4. Statistical Optimization of Gibberellic Acid Production by Box-Behnken Design

Based on the results of Taguchi methods, four factors (date waste, NaNO_3_, urea, and (NH_4_)_2_SO_4_) were found to have a greater influence on GA3 production by the new isolate strain B28. Afterwards, the Box-Behnken design was used to optimize the level of each of these factors giving the highest production and to study their interaction. Experimental conditions and results for GA3 yield are presented in Table 5.

The calculated regression analysis indicated that the *F* value was 9.745, with a very low probability value (*P* < 0.0001) showing the significance of the model. The closeness of experimental and predicted GA3 production can be expressed by the determination coefficient (*R*^2^ = 0.78) which stipulates that only 22% of the total variation could not be explained by the model. Adjusted R Square of 0.70 indicated that the regression model could be used to analyze trends of responses. The analyses of the quadratic model showed that urea present the largest effect on GA3 production in SSF. This variable shows a significant positive linear effect (*X*3) and an important negative quadratic term (*X*3*X*3). The linear and the quadratic terms of the other variables as well as the first-order interaction between the tested factors did not show any major significant effect. These results were confirmed by Student's *t* test (*α* = 0.05) (Table 6). The chosen model equation showed that the predictive model was selected based on the highest Adjusted R Square. Also, we noticed that the linear term of (NH_4_)_2_SO_4_ is eliminated from this model, which means that this factor did not have a first-order significant effect on the GA3 production. These results are in good agreement with the studies done by Panchal and Desai [26] who tested different nitrogen sources (NH_4_Cl, NH_4_NO_3_, (NH_4_)_2_SO_4_, (NH_4_)MoO_4_, and urea), as an additional substrate of commercial wheat bran, on the *Fusarium moniliforme* GA3 production. They found that urea had a remarkable effect on GA3 production and demonstrated that urea exhibits buffering activity and thus resists the change in pH during fermentation. The authors also noted that the lower production levels of GA3 with ammonium salts may be due to the decrease in pH with the utilization of NH_4_ ions.

The data indicated that carbon and nitrogen sources can act as nutrients or limiting factors. In fact, a slight change in their concentrations can lead to a significant variation in the production level. An increase that reached up to 7.95 g/flask in the GA3 production in SSF was recorded with the increase of NaNO_3_ concentration to 0.75 g/flask and the decrease of date waste concentration to 5 g/flask. Production levels (6.37 g/flask) were noted to increase with the decrease of (NH_4_)_2_SO_4_ and date waste concentrations. Rodrigues et al. [30] reported that the quality and quantity of nitrogen sources are very important factors for the gibberellin production due to the ammonium regulation of the process. They noted that the higher GA3 yields are reached when the nitrogen concentration is low in the media. The GA3 production begins when nitrogen is exhausted [31, 32].

Under the optimized culture media, the quadratic model showed that the maximum GA3 production would be 8.29 g/flask, when date waste, NaNO_3_, urea, and (NH_4_)_2_SO_4_ were 5, 0.75, 0.19, and 0.25 g/flask, respectively. To validate the predicted results, fermentation experiments were performed in two tests. GA3 production yield (8.16 g/flask) was absolutely more important than that obtained during the initial study (2.72 g/flask). Thus, the production level was multiplied by a factor of 3. In this study, the successive optimization strategies (Plackett-Burman, L25 Taguchi method, and Box-Behnken design) were successfully applied to test the relative importance of medium components in the GA3 production. The finding indicates that, after optimization of the culture medium, the production levels of GA3 by *Fusarium oxysporum* were reached before (Table 7).

### 3.5. Effects of GA3 Exogenous Application on Tomato

In the current experiments, salt stress conditions induced a reduction (*P* < 0.05) in morphological traits such as the plant height, number of leaves and fruits per plant, and fresh and dry plant weight (Table 8). The reduction in the numbers of leaves and fruits per plant in salt condition was, respectively, 33.6 and 45.8% in SSP1 and 46.9 and 66.6% in SSP2. The inhibition of growth characteristics of tomato plants were significantly alleviated (*P* < 0.05) by the GA3 foliar spray application. In fact, the exogenous application of GA3 using different concentrations (10^−5^, 10^−6^, and 10^−7^ M) increased shoot length, leaf and fruit numbers, and fresh and dry shoot weights. Interestingly, maximum values of growth traits were obtained with the GA3 level of 10^−6^ M (GA32). The SSP1+GA32 and SSP2+GA32 treated plants showed an improvement (*P* < 0.05) by 1.67 and 1.32 times, respectively, when compared to the untreated plants with GA3 (SSP1 and SSP2). The utilization of GA3 crude extract obtained by SSF to ameliorate the adverse salinity effect on tomato plants (*Solanum lycopersicum* L.) is being reported for the first time. Several reports showed that the exogenous application of GA3 alleviates the adverse effects of salinity stress on plant growth [33, 34]. Maggio et al. [35] reported that in tomato plants, exogenous application of GA3 increased morphological traits and improved the yield.

**Figure 2:**
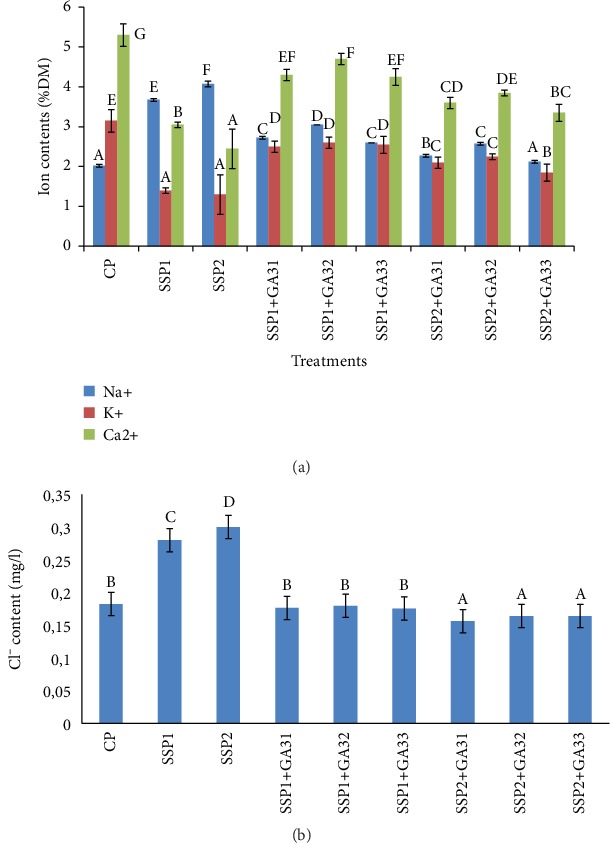
(a) Accumulation of Na^+^, K^+^, and Ca^2+^ in leaves of tomato plants under saline conditions and exogenous gibberellic acid. (b) Accumulation of Cl^−^ in leaves of tomato plants under saline conditions and exogenous gibberellic acid. The different letters (A, B, C, D, and E) indicate significant differences among treatments (*P* < 0.05) according to Tukey's test.

Under saline conditions, toxic ions such as chloride and sodium were accumulated in the tissue of the majority of plants [36, 37]. In fact, osmotic adjustment is completed by ion uptake or through the accumulation of compatible solutes. The effect of salt stress on macronutrient content of tomato plants is presented in Figures 2(a) and 2(b). The finding revealed that in SSP1 and SSP2 treated plants, salinity was shown to increase the Na^+^ and Cl^−^ levels and to reduce the K^+^ and Ca^2+^ levels, however, exogenous application of GA3 resulted in a significant improvement of Ca^2+^ and K^+^ contents (Figure 2(a)) and an important reduction (*P* < 0.05) of Na^+^ and Cl^−^ contents (Figure 2(b)) in the leaves of tomato plants. As compared to the SSP2-treated plants, the K^+^ and Ca^2+^ contents of SSP2+GA32-treated plants were, respectively, increased by 42 and 36%, and the contents of Na^+^ and Cl^−^ were reduced by 44 and 45%, respectively. These results agree with Tuna et al. [38], who reported that GA3 reduced the accumulation of Na^+^ and enhanced K^+^ and Ca^2+^ levels in leaves of maize plants under saline conditions. The characteristics of tomato plants grown under salt stress were effectively improved by the GA3 application. It was indicated that Gibberellic acid was useful to enhance ion contents of many plants under stress conditions, such as tomato, wheat, and maize [35, 39, 40].

**Figure 3:**
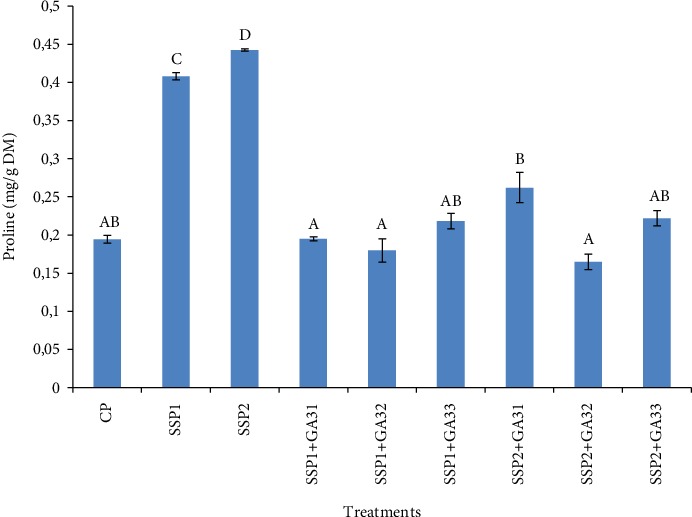
The effect of salt stress and exogenous application of GA3 on proline content in tomato leaves. The different letters (A, B, C, D, and E) indicate significant differences among treatments (*P* < 0.05) according to Tukey's test.

In this study, we also determined the proline contents in plants untreated and treated with GA3 under salt stress conditions (Figure 3). The data showed that proline content increased in leaves with the increment of salt stress. In fact, in SSP2-treated plants, proline content was about 0.44 mg/g DM in leaf tissues. The accumulation of proline, an amino acid protectant, was reported in olive plants (*Olea europaea*) exposed to salt stress [41, 42]. These reports suggested that proline is also a metabolite of adaptation to the salinity. It was reviewed that under salinity stress, high levels of proline in leaves of various plants could be explained by gene expressions encoding enzymes of proline synthesis (pyrroline-5-carboxylate) or by a decrease in proline enzymes such as oxidation proline dehydrogenase [43]. Indeed, proline accumulation in plant tissues is an adaptive strategy in stressful environments, which maintains the osmotic balance, stabilizes cell membrane structure, and regulates cellular redox potential [6, 44–46]. The obtained results (Figure 3) also revealed that exogenous *F. oxysporum* GA3 application decreased (*P* < 0.05) proline content under different salinity treatments. In fact, in SSP1+GA32-treated plants, this decrease was about 35% in leaves of tomato plants in comparison to the values recorded in SSP1-treated plants (0.4 mg/g DM). These results were in agreement with the study of Tuna et al. [38], who noted that the exogenous application of GA3 alleviates the adverse effect of NaCl by decreasing the proline content in maize plants.

**Figure 4:**
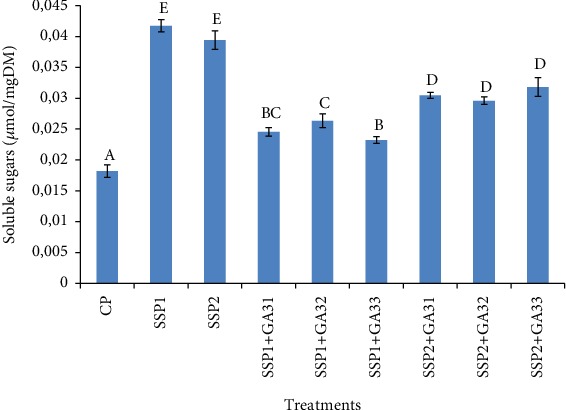
Accumulation of soluble sugar content in leaves of tomato plants under saline conditions and exogenous gibberellic acid. The different letters (A, B, C, D, and E) indicate significant differences among treatments (*P* < 0.05) according to Tukey's test.

Solutes, such as sugars, accumulate in the cytosol under salt stress and can therefore contribute to plant survival. They play an important role in the osmoregulation under several conditions [47]. In this study, leaves of tomato plants exposed to two salt stress treatments accumulated high soluble sugar contents (*P* < 0.05) as compared to the unstressed plants (CP). It was about 41.1 (mol/g DM) in SSP1 and 38.5 (mol/g DM) in SSP2. However, the exogenous application of *F. oxysporum* GA3 at both levels led to the decrease of soluble sugars contents (*P* < 0.05) in leaf tissues of tomato plants (Figure 4). Indeed, the reduction of soluble sugars contents, in the presence of 10^−6^ M GA3, was 1.6 and 1.2-fold less than SSP1 and SSP2-treated plants, respectively. These results are in accordance with those obtained by Iqbal et al. [43].

**Figure 5:**
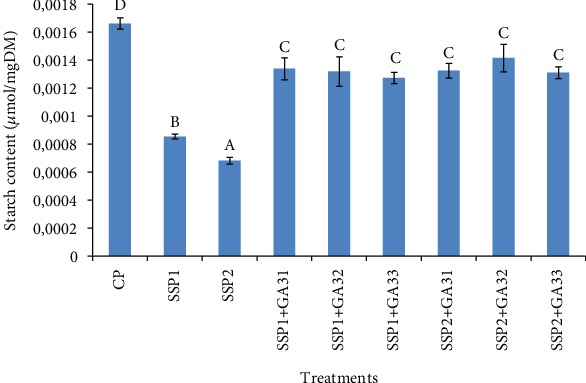
The effect of salt stress and exogenous application of GA3 on starch content in tomato leaves. The different letters (A, B, C, D, and E) indicate significant differences among treatments (*P* < 0.05) according to Tukey's test.

Data presented in Figure 5 show that starch content in leaves of tomato plants were significantly reduced (*P* < 0.05) by both salinity treatments, when compared to the unstressed control plants. The lowest values of starch contents were recorded in SSP2-treated plants (0.67 (mol/g DM)). The externally applied GA3 increased the starch content in treated plants, but the levels were still lower than those of control plants (1.65 (mol/g DM)). Under stressed conditions, the highest amounts of starch content in leaf tissues were recorded in SSP1+GA32-treated plants.

The higher accumulation of sugars and proline in leaves (*P* < 0.05), compared to unstressed plants, could be due to their photosynthetic activity. In fact, leaves of tomato plants with low photosynthetic activity tend to synthesize more soluble sugars. Furthermore, the decrease in starch content in plants under stressed conditions could be due to starch degradation and/or to the increment in soluble sugar concentration under limited water availability. Todaka et al. [48] demonstrated that in stressed plants, *β*-amylase activity increased. The lower starch concentrations in leaves of salt-stressed plants suggest that carbon was translocated out of the leaves.

**Figure 6:**
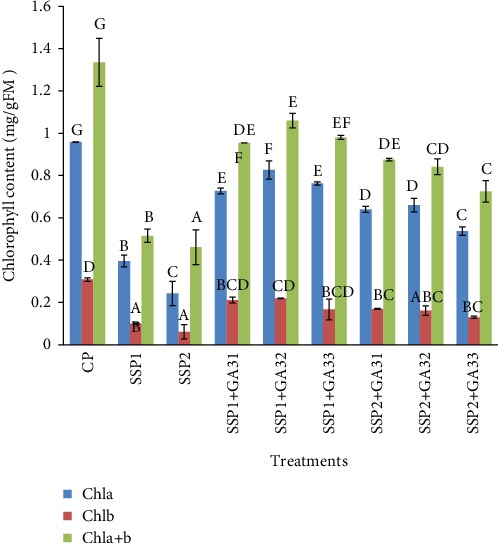
Dynamic variation of chlorophyll results of tomato leaves under salt stress conditions and GA3 exogenous application. The different letters (A, B, C, D, and E) indicate significant differences among treatments (*P* < 0.05) according to Tukey's test.

Salt stress disturbs several aspects of plant mechanisms, such as photosynthesis and pigment synthesis [49]. The data in Figure 6 show that 50 and 100 mM NaCl treatments caused a significant decrease (*P* < 0.05) of chlorophyll (a, b, and a+b) content, in comparison to control plants. The GA3 treatment increased (*P* < 0.05) the contents of photosynthetic pigments (Figure 6). The highest levels were recorded in SSP1+GA32-treated plants. These values were 0.82, 0.21, and 1.06 mg/g of FM, for chlorophyll (a, b, and a+b) contents, respectively. In the case of soybean plants, Zhao et al. [50] reported that treatment with GA3 increased pigments content. Also, it has been demonstrated that chlorophyll and carotenoids contents of maize leaves were increased by the treatment with GA3 [51].

## 4. Conclusion

The endophytic fungus *F. oxysporum*, isolated from root of flowers plant, showed a good ability to produce maximal yield of gibberellic acid on low-cost substrate under SSF. Under the optimization, the maximal yield of GA3 (8.16 g/kg ds) increased about 2.72-folds from the initial production medium. In the present work, the application of GA3 at 10^−6^ M provided an efficient regulator for tomato plants under salt stress.

## Figures and Tables

**Table 1:** Variables and nutrient screened on the Plackett–Burman design in gibberellic acid production by *F. oxysporum*.

Run	Global	Inoculum size (%)	Wheat bran (g/flask)	Sesame bark (g/flask)	Wheat straw (g/flask)	Barley bran (g/flask)	Sucrose (g/flask)	Date waste (g/flask)	Molasse (g/flask)	NaNO_3_ (g/flask)	Fish meal (g/flask)	Urea (g/flask)	NH_4_NO_3_ (g/flask)	(NH_4_)_2_SO_4_ (g/flask)	Yield (g/kg ds)
1	1	10	5	0	0	10	3	0	3	0.5	0	0.1	0	0.5	3.041
2	1	10	10	0	0	0	10	0	0	0.5	0.5	0	0.1	0	1.008
3	1	10	10	10	0	0	3	5	0	0	0.5	0.1	0	0.5	2.606
4	1	10	10	10	10	0	3	0	3	0	0	0.1	0.1	0	2.166
5	1	5	10	10	10	10	3	0	0	0.5	0	0	0.1	0.5	2.339
6	1	10	5	10	10	10	10	0	0	0	0.5	0	0	0.5	1.364
7	1	5	10	0	10	10	10	5	0	0	0	0.1	0	0	1.483
8	1	10	5	10	0	10	10	5	3	0	0	0	0.1	0	0.830
9	1	10	10	0	10	0	10	5	3	0.5	0	0	0	0.5	2.319
10	1	5	10	10	0	10	3	5	3	0.5	0.5	0	0	0	1.986
11	1	5	5	10	10	0	10	0	3	0.5	0.5	0.1	0	0	1.938
12	1	10	5	0	10	10	3	5	0	0.5	0.5	0.1	0.1	0	3.009
13	1	5	10	0	0	10	10	0	3	0	0.5	0.1	0.1	0.5	1.644
14	1	5	5	10	0	0	10	5	0	0.5	0	0.1	0.1	0.5	4.274
15	1	5	5	0	10	0	3	5	3	0	0.5	0	0.1	0.5	1.991
16	1	5	5	0	0	0	3	0	0	0	0	0	0	0	1.242
BJ	1, 90	-0, 03	-0, 13	0, 11	-0, 0014	-0, 11	-0, 22	0, 23	-0, 08	0, 41	-0, 13	0, 44	0, 08	0, 36	

**Table 2:** Natural level and effect of significant independent variables on the gibberellic acid production by *F. oxysporum* strain using the Plackett-Burman design.

Variable code	Variables	Unit	Level		*b*	*t* value	*P* > ∣t∣
			(−1)	(+1)			
*X*3	Sesame bark	(g/flask)	0	10	10.968	2.012	0.182
*X*4	Wheat straw	(g/flask)	0	10	9.615	1.763	0.220
*X*7	Date waste	(g/flask)	0	5	10.226	1.876	0.202
*X*9	NaNO_3_	(g/flask)	0	0.5	15.020	2.755	0.101
*X*11	Urea	(g/flask)	0	0.1	12.086	2.215	0.157
*X*13	(NH_4_)_2_SO_4_	(g/flask)	0	0.5	10.372	1.902	0.197

*b*: unstandardized coefficients. *P* > ∣*t*∣: significance.

**Table 3:** Factors and their levels as studied by the orthogonal array of Taguchi L25 design for optimization of gibberellic acid production by *F. oxysporum*.

Run	Sesame bark	Wheat straw	Date waste	NaNO_3_	Urea	(NH_4_)2SO4	Yield (g/kg ds)
1	1	1	1	1	1	1	2.831
2	1	2	2	2	2	2	3.329
3	1	3	3	3	3	3	2.561
4	1	4	4	4	4	4	2.519
5	1	5	5	5	5	5	2.706
6	2	1	2	3	4	5	2.961
7	2	2	3	4	5	1	5.388
8	2	3	4	5	1	2	4.184
9	2	4	5	1	2	3	4.712
10	2	5	1	2	3	4	2.156
11	3	1	3	5	2	4	2.884
12	3	2	4	1	3	5	4.055
13	3	3	5	2	4	1	3.309
14	3	4	1	3	5	2	5.045
15	3	5	2	4	1	3	2.822
16	4	1	4	2	5	3	2.166
17	4	2	5	3	1	4	1.726
18	4	3	1	4	2	5	2.549
19	4	4	2	5	3	1	2.834
20	4	5	3	1	4	2	2.710
21	5	1	5	4	3	2	6.349
22	5	2	1	5	4	3	2.484
23	5	3	2	1	5	4	3.036
24	5	4	3	2	1	5	2.851
25	5	5	4	3	2	1	2.672
							

**Table 4:** Main effects of factor, difference of levels (2) and (1), and contribution (%) and optimum values of factors.

Nutriment code	Level (1)^a^	Level (2)^a^	Level (3)^a^	Level (4)^a^	Level (5)^a^	L2-L1^b^	Optimum values	Contribution
Sesame bark	2.789	3.88	3.62	2.40	3.48	1.09	0.65	17%
Wheat straw	3.438	3.40	3.13	3.59	2.61	-0.04	0.36	10%
Date waste	3.013	3.00	3.28	3.12	3.76	-0.02	0.53	14%
NaNO_3_	3.469	2.76	2.99	3.93	3.02	-0.71	0.69	18%
Urea	2.883	3.23	3.59	2.80	3.67	0.35	0.43	12%
(NH_4_)2SO4	3.407	4.32	2.95	2.46	3.03	0.92	1.09	29%

^a^Value is given in terms of production yield (g/kg ds). ^b^Production yield difference between level 2 and level 1.

**Table 5:** The Box-Behnken design for the four independent variables.

Run	Date waste	NaNO_3_	Urea	(NH_4_)_2_SO_4_	Yield (g/kg ds)
1	5	0.25	0.2	0.5	6.867
2	5	0.75	0.2	0.5	7.956
3	15	0.25	0.2	0.5	8.168
4	15	0.75	0.2	0.5	5.580
5	10	0.5	0.1	0.25	6.554
6	10	0.5	0.1	0.75	5.870
7	10	0.5	0.3	0.25	6.366
8	10	0.5	0.3	0.75	6.051
9	5	0.5	0.2	0.25	7.381
10	5	0.5	0.2	0.75	6.524
11	15	0.5	0.2	0.25	6.526
12	15	0.5	0.2	0.75	6.136
13	10	0.25	0.1	0.5	6.529
14	10	0.25	0.3	0.5	5.573
15	10	0.75	0.1	0.5	6.498
16	10	0.75	0.3	0.5	5.928
17	5	0.5	0.1	0.5	6.177
18	5	0.5	0.3	0.5	6.619
19	15	0.5	0.1	0.5	6.604
20	15	0.5	0.3	0.5	6.521
21	10	0.75	0.2	0.25	7.379
22	10	0.75	0.2	0.75	6.451
23	10	0.75	0.2	0.25	6.746
24	10	0.75	0.2	0.75	6.527
25	10	0.5	0.2	0.5	7.297
26	10	0.5	0.2	0.5	6.984
27	10	0.5	0.2	0.5	7.036

**Table 6:** Analysis of the model terms by Student's *t* test.

Coefficients	Nonstandardized coefficients		Standardized coefficients	*t*	Significance
	*B*	Beta	Beta		
*X*1	2.004	0.931	0.000	2.153	0.044
*X*2	0.261	0.093	1.410	2.823	0.011
*X*3	6.847	1.437	1.801	4.763	0.000
*X*1*X*2	26.871	5.549	2.900	4.843	0.000
*X*1*X*4	-0.735	0.138	-2.829	-5.347	0.000
*X*3*X*3	0.146	0.118	0.569	1.243	0.229
*X*4*X*4	-69.624	13.649	-3.057	-5.101	0.000

**Table 7:** GA3 production by solid state fermentation in the literature.

Substrates	Nitrogen source	Fermentation technique	Production	Reference
Coffee husk cassava bagasse	(NH_4_)_2_SO_4_	SSF	492.5 mg of GA3/kg	Machado et al. [27]
Citric pulp		SSF	5.9 g/kg of dry citric pulp	Rodrigues et al. [28]
Pigeon pea pod	NH_4_NO_3_	SSF	7.8 g/kg of dry substrate	Satpute et al. [29]
Commercial wheat bran	NH_4_Cl, NH_4_NO_3_, (NH_4_)_2_SO_4_, (NH_4_)MoO_4_	SSF	1160 *μ*g/g of dry substrate	Panchal and Desai [26]
Citric pulp and soy husk	(NH_4_) NO_3_, (NH_4_)_2_ SO_4_, urea	SSF	5.9 g/kg of dry substrate	Rodrigues et al. [52]
Citric pulp		SSF	7.60 g/kg of dry substrate	Oliveira et al. [24]

**Table 8:** The effect of saline condition and exogenous gibberellic acid on growth parameters of tomato plants.

Treatments	Plant height (cm)	Leaf number per plant	Fruit number per plant	Fresh material (g/plant)	Dry material (g/plant)
CP	69.6 ± 3.05^d^	108 ± 6.08^d^	8 ± 1^d^	76.5 ± 2.33^e^	11.03 ± 1.0^e^
SSP1	46 ± 1.74^b^	71.66 ± 3.51^b^	4.33 ± 0.57^abc^	51 ± 1.56^ab^	7.46 ± 0.15^bc^
SSP2	36.43 ± 0.81^a^	57.33 ± 1.52^a^	2.66 ± 0.57^a^	45.3 ± 2.53^a^	6 ± 0.17^a^
SSP1+GA31	57.7 ± 2.68^c^	90.33 ± 5.50^c^	6 ± 1^cd^	55.93 ± 1.48^bc^	8.1 ± 0.17^c^
SSP1+GA32	62.86 ± 3.47^c^	98.33 ± 6.11^cd^	6.33 ± 0.57^cd^	66.46 ± 3.81^d^	9.56 ± 0.20^d^
SSP1+GA33	58 ± 1.11^c^	90 ± 1^c^	5 ± 1^bc^	59.36 ± 1.09^c^	8.36 ± 0.37^c^
SSP2+GA31	42.9 ± 1.9^b^	67.33 ± 3.21^ab^	2.66 ± 0.57^a^	54.16 ± 0.76^bc^	7.56 ± 0.15^bc^
SSP2+GA32	48.1 ± 1.85^b^	73 ± 2.64^b^	3.33 ± 0.57^ab^	59.86 ± 3.47^c^	8.16 ± 0.15^c^
SSP2+GA33	37.73 ± 2.05^a^	59 ± 5.29^a^	3 ± 1^ab^	44.8 ± 0.45^a^	6.66 ± 0.20^ab^

± Standard error. Different letters in the same column are significantly different (*P* < 0.05).

## Data Availability

The datasets generated and/or analyzed during the current study are available on the GenBank repository, https://www.ncbi.nlm.nih.gov/genbank/. The GenBank accession number for the nucleotide sequence of the 5.8 Its gene referred to in the text is MN816007. Other datasets generated during and/or analyzed during the current study are available from the corresponding author on reasonable request.

## Abbreviations

(NH_4_)_2_SO_4_: Ammonium sulfate
CWB: Commercial wheat bran
DM: Dry matter
FeSO4: Ferrous sulfate
FM: Fresh matter
GA3: Gibberellic acid
H_2_SO_4_: Sulfuric acid
HCL: Chlorhydric acid
HNO_3_: Nitric acid
ITS: Internal transcribed spacer
K_2_HPO_4_: Dipotassium phosphate
KCl: Potassium chloride
MeOH: Methanol
MgSO_4_.7H_2_O: Magnesium sulfate heptahydrate
NaCl: Sodium chloride
NaNO_3_: Sodium nitrate
NH_4_NO_3_: Ammonium nitrate
PCR: Polymerase chain reaction
PDA: Potato dextrose agar
SMF: Submerged fermentation
SSF: Solid state fermentation.
